# Assessment of Motor Control during Three-Dimensional Movements Tracking with Position-Varying Gravity Compensation

**DOI:** 10.3389/fnins.2017.00253

**Published:** 2017-05-16

**Authors:** Yao Huang, Qianqian Yang, Ying Chen, Rong Song

**Affiliations:** ^1^Key Laboratory of Sensing Technology and Biomedical Instrument of Guangdong Province, School of Engineering, Sun Yat-sen UniversityGuangzhou, China; ^2^Guangdong Provincial Engineering and Technology Center of Advanced and Portable Medical Devices, School of Engineering, Sun Yat-sen UniversityGuangzhou, China

**Keywords:** gravity compensation, upper limb rehabilitation, cable-based rehabilitation robotics, arm tracking, muscle activation

## Abstract

Active movements are important in the rehabilitation training for patients with neurological motor disorders, while weight of upper limb impedes movements due to muscles weakness. The objective of this study is to develop a position-varying gravity compensation strategy for a cable-based rehabilitation robot. The control strategy can estimate real-time gravity torque according to position feedback. Then, the performance of this control strategy was compared with the other two kinds of gravity compensation strategies (i.e., without compensation and with fixed compensation) during movements tracking. Seven healthy subjects were invited to conduct tracking tasks along four different directions (i.e., upward, forward, leftward, and rightward). The performance of movements with different compensation strategies was compared in terms of root mean square error (RMSE) between target and actual moving trajectories, normalized jerk score (NJS), mean velocity ratio (MVR) of main motion direction, and the activation of six muscles. The results showed that there were significant effects in control strategies in all four directions with the RMSE and NJS values in the following order: without compensation > fixed compensation > position-varying compensation and MVR values in the following order: without compensation < fixed compensation < position-varying compensation (*p* < 0.05). Comparing with movements without compensation in all four directions, the activation of muscles during movements with position-varying compensation showed significant reductions, except the activations of triceps and in forward and leftward movements, the activations of upper trapezius and middle parts of deltoid in upward movements and the activations of posterior parts of deltoid in all four directions (*p* < 0.05). Therefore, with position-varying gravity compensation, the upper limb cable-based rehabilitation robotic system might assist subjects to perform movements with higher quality and improve the participation of robot-aided rehabilitation training. Further studies are needed to explore the effectiveness and clinic application across pathologies.

## Introduction

Since the upper limb dysfunctions after stroke seriously affect daily lives, it is essential for patients to restore the affected motor functions through rehabilitation training. Post-stroke reaching movements is affected by gravity weight of upper limb due to the weakness of those anti-gravity muscles (Beer et al., 2007). Position control was adopted in traditional robots (Lahouar et al., 2009; Yuan et al., 2014) and rehabilitation robots (Lum et al., 2002; Staubli et al., 2009), to follow predetermined trajectories. Although, position control in rehabilitation robots could counteract the effect of gravity weight of upper limb, it focused on control accuracy and ignores patients' voluntary participation in task execution, and its effectiveness in rehabilitation therapy needed further improvement. For patients who suffer from muscle weakness, gravity support showed promising results in minimizing the gravity-induced interference for tasks execution (van Elk et al., 2005; Cheng et al., 2015; Runnalls et al., 2015).

Gravity compensation can be provided by support devices (Herder et al., 2006; Kloosterman et al., 2010) or robotics (Kahn et al., 2006; Ball et al., 2007; Ladenheim et al., 2013), and can be generally grouped into three categories. Firstly, fixed or manually adjusted compensation were commonly used to counteract the gravity weight of upper limb (Nef et al., 2007; Stopforth, 2013; Lenzo et al., 2015). Similarly, a fixed external vertical force was applied to an upper limb robot by a motorized vertical cabling system for gravity compensation (Ball et al., 2007). Secondly, passive compensations by flexible force from elastic materials were chosen (De Luca et al., 2005; Stienen et al., 2009). As an assistive arm exoskeleton, T-WREX provided gravity compensation from the elastic bands whose number could be adjusted at different arm support levels (Housman et al., 2009). Arm orthosises with springs was designed to passively compensate arm weight, and could help patients with little moving ability to reach and grasp (Herder et al., 2006; Kramer et al., 2007). Freebal, a device with gravity compensation at different levels from cables connected to springs, could facilitate upper limb movements tracking in the horizontal and vertical planes (Prange et al., 2009; Kloosterman et al., 2010; Coscia et al., 2014). Thirdly, position-varying compensations can be provided according to human physical characteristics, since gravity torque of upper limb is highly coupled with the dynamics of the limbs and dependent on the postures and positions of moving limbs. Hsu et al. proposed an active control strategy to estimate the subject's movement intention and the control strategy included a gravity compensation term modified by the upper limb dynamics (Hsu et al., 2012). Cheng et al. developed a two degrees of freedom (2-DOF) compliant beam which could compensate the torques on each joint based on the dynamic of the upper limb (Cheng et al., 2015). A torque-angle model containing a gravity compensation term was proposed by Lin et al. to evaluate motion quality of adhesive capsulitis patients (Lin et al., 2014).

Since the gravity torque is highly dependent on positions of upper and fore arm during movements, fixed or varying elastic compensation strategies did not consider the position-coupling effect. Although, Hsu et al. and Li et al. included compensation strategies according to human physical characteristics, both their strategies were combined with other control terms (Hsu et al., 2012; Li et al., 2015) which might affect voluntary participation. Gravity compensation should provide suitable assistance to subjects without affecting voluntary participation, but few previous studies about compensation strategy considered both varying position of upper limb and voluntary participation. Meanwhile, how varying position affects movement performance and muscle activations is rarely reported.

The objective of this study is to develop a position-varying gravity compensation strategy for a cable-based rehabilitation robot. A gravity torque estimation model according to position feedback is proposed for estimating real-time upper limb gravity torque. Then, the performance of this strategy is compared with the other two kinds of gravity compensation strategies (i.e., without compensation and with fixed compensation) during four different directions of man-machine cooperation movements tracking. Root mean square error (RMSE) between target and actual moving trajectories, normalized jerk score (NJS), mean velocity ratio (MVR) of main motion direction, and the mean activation of six muscles are used for assessing different compensation strategies with a cable-based rehabilitation robot.

## Materials and methods

### Participants

Seven healthy men (mean age: 23.7 ± 1.1 yrs., mean weight: 63.5± 9.3 kg, mean height: 172.5 ±4.5 cm) were recruited in this study. All the subjects were able to lift their right arm against gravity, and had no musculoskeletal or neurological problems. All subjects provided their written informed consent prior to participating in this study. All experimental procedures were approved by the human ethic committee of the First Affiliated Hospital of Sun Yat-sen University.

### Experimental apparatus

**Figure 1:**
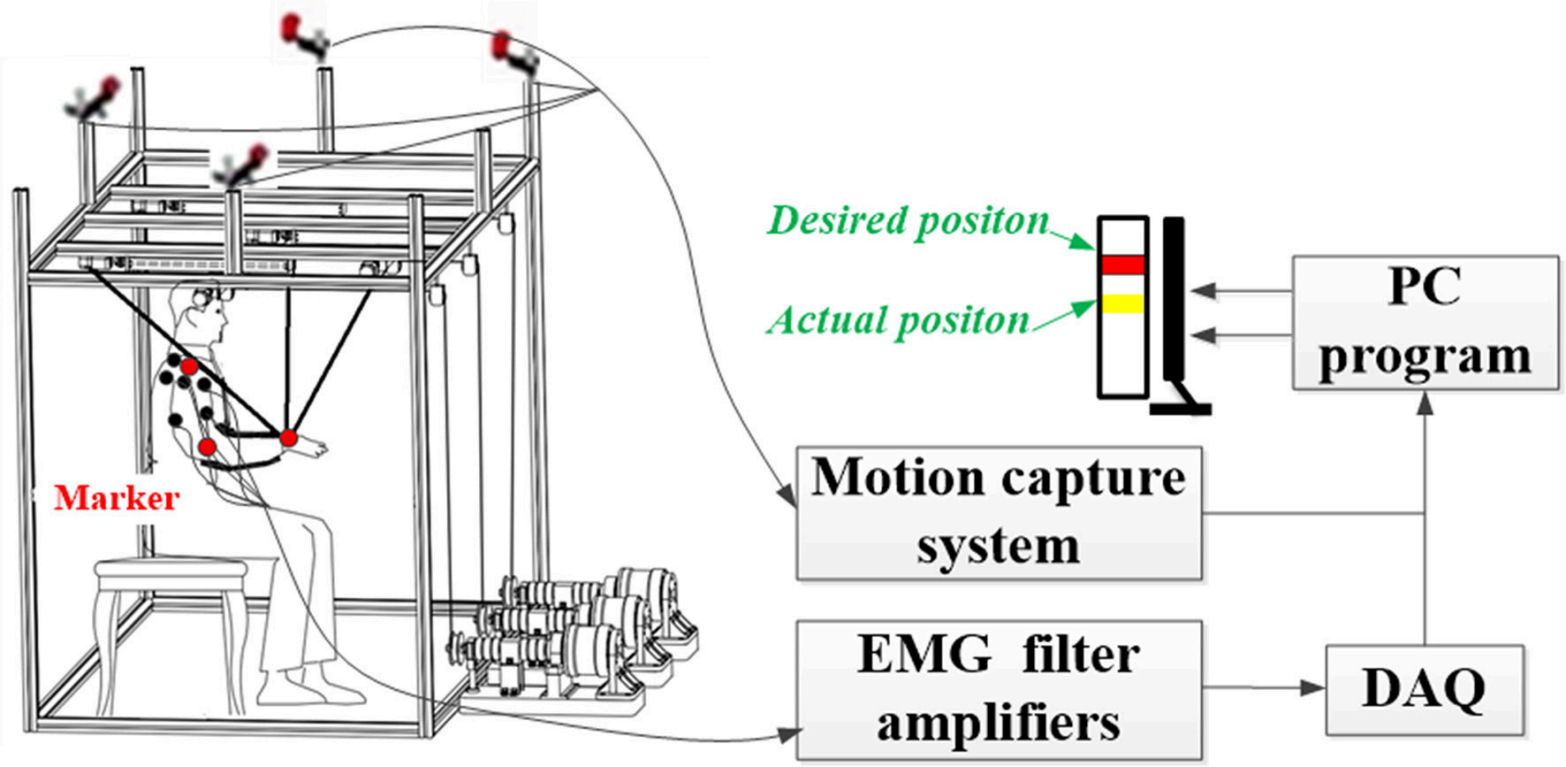
**Architecture of the cable-based rehabilitation robot**.

A cable-based rehabilitation robotic system was recruited for providing assistance to counteract the influence of gravity during multi-joints upper limb movements (Figure 1). The robotic system consisted of a mechanical part, a motion capture system with four cameras (OptiTrack, NaturalPoint, USA), a six-channel surface electromyographic (EMG) signals amplifier, a 16-bit analog-to-digital data acquisition card (PXI-6229, National Instruments, USA), and a personal computer. The mechanical part composed of a cubic base frame made of aluminum links, a splint, three cables, and three motors (DM1B-045G, Yokogawa, Japan) with three servo drivers (UB1DG3, Yokogawa, Japan; Yang et al., 2016). The splint was controlled by three motors through three bundled cables, resulting in 3 degrees of freedom (DOF). The group of motors could apply force on the split and assist the user performing movements in a 3-dimensional (3D) space. Three markers were attached at the dorsal centers of three joints (i.e., wrist, elbow, and shoulder), respectively, to record actual positions by the motion capture system. The sampling rate of the motion capturing was set at 100 Hz, and raw position data were filtered with a second-order Butterworth filter with a cut-off frequency of 6 Hz. Bi-polar surface EMG of six superficial muscles of the upper extremity [i.e., biceps (BIC), triceps (TRI), anterior (DA), middle (DM), posterior (DP) parts of deltoid, and upper trapezius (TRA)] were recorded by attaching electrodes on the subject's skin. The raw EMG data from the six muscles were converted by the data acquisition card. The EMG signals were recorded at 1,000 Hz, amplified with a gain of 5,000, and band-filtered by a 4th-order Butterworth filter with a band of 10–400 Hz.

### Experimental procedure

**Figure 2:**
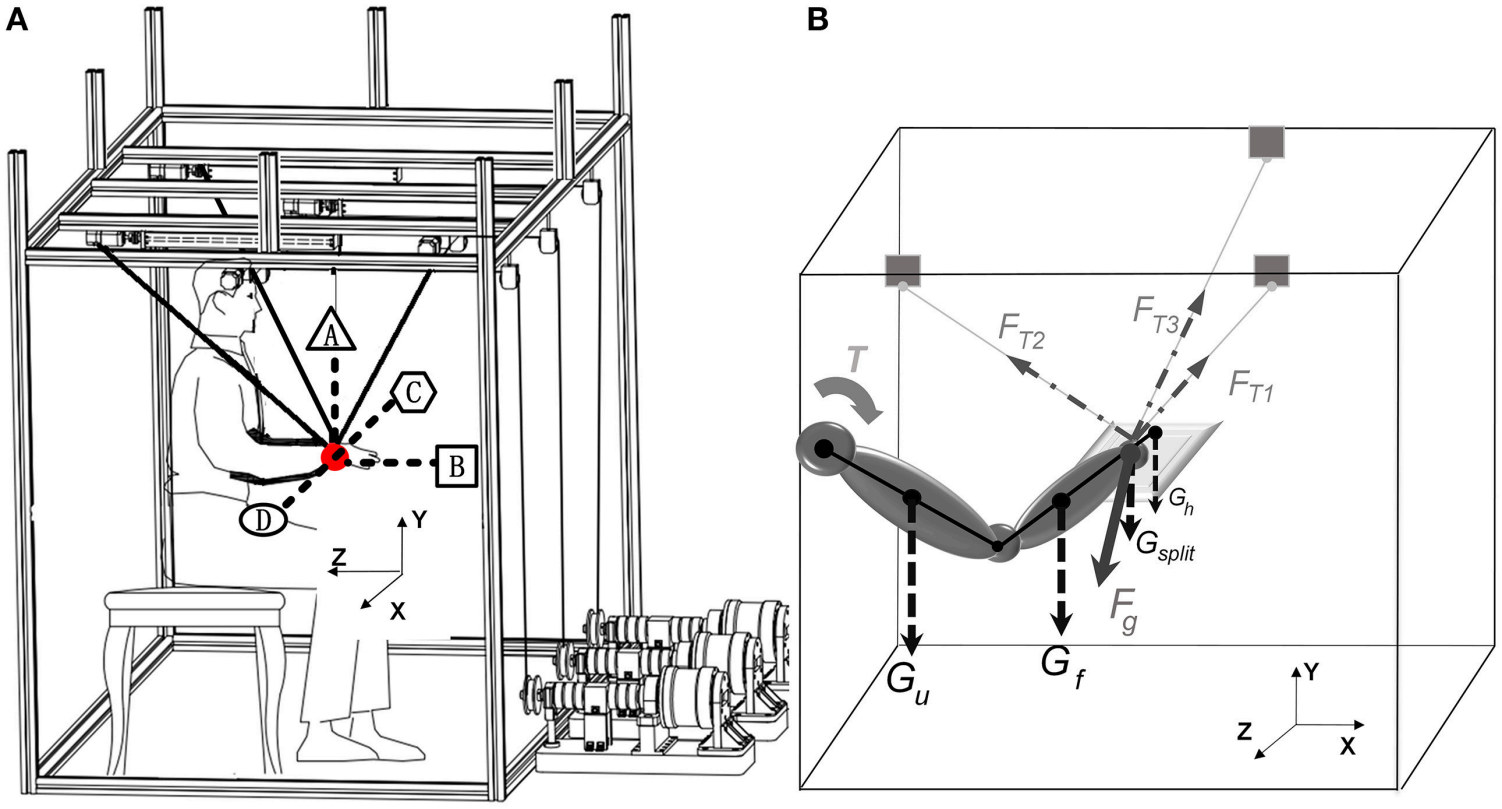
**(A)** The tasks directions and **(B)** static force model.

Before experiment, subjects should be seated in a chair and their trunks were strapped by soft fabrics to minimize the trunk movements. The initial posture of upper limb positioned at: the elbow flexion was at 90°, the forearm pronation was at 90°, the upper arm hung down vertically and was close to the trunk, the wrist was extended and the fingers were closed. A computer screen was placed in front of the subject for providing real-time visual feedback of the target and actual wrist positions. A yellow slide block represented the user's wrist showed the actual wrist position, and a red sliding block represented the target position. As shown in Figure 2A, each subject went through four kinds of multi-joint tracking tasks with the right arm [i.e., move upward (A)/forward (B)/leftward (C)/rightward (D) to the place 0.2 m away from the initial point along a straight line]. During one movement series, subjects are asked to finish three movement sessions including three kinds of gravity compensation strategies. And each session required the execution of six consecutive laps.

### Gravity compensation strategies

The position-varying gravity compensation strategy was based on real-time estimation of upper limb gravity torque. A gravity torque estimation model was built to estimate the gravity torque from the joint positions and moments of the shoulder, elbow, and wrist:

(1) $$T=G_{u}L_{ush}\;+\;G_{f}L_{fsh}+G_{h}L_{hsh}$$

where ***T*** was the gravity torque of upper limbs about shoulder,***G_h_***, ***G_f_***, ***G_u_*** were the gravity of hand, forearm and upper arm, respectively, and *L*_*hsh*_, *L*_*fsh*_, *L_ush_* were the moment arms from limb centroids of hand, forearm and upper arm to centroid of shoulder joint, respectively.

The equivalent force to the wrist can be calculated from the gravity torque as Equation (2):

(2) $$F_{g}=T/L_{shw}$$

where ***F_g_*** here was the equivalent force of the arm, *L*_*shw*_ was moment arm from limb centroids of shoulder to centroid of wrist joint.

The dynamic characteristics of the robotic system can be analyzed based on the static force model when the splint moved slowly and stably (Figure 2B). The resultant compensation force (*F*_*OUT*_) then can be expressed as Equation (3)

(3) $$F_{OUT}=-(F_{g}+G_{split})$$

where ***G_split_*** was the gravity of the split.

Orientations of the three cables should be considered when driving the motors and controlling the split. The cable-based structure required all cables kept in tension, and the tensile forces needed during arm movements can be presented as Equation (4):

(4) $$F_{T}=J^{-1}F_{OUT}$$

where ***F*_*T*_** = [***F*_*T*1_**, ***F*_*T*2_**, ***F*_*T*3_**]^***T***^ was the tensile force matrix, and *J* = [**u**_1_, ***u***_2_, ***u***_3_] was the unit vector matrix of the mechanism structure.

There were the other two kinds of gravity compensation strategies (i.e., without compensation and with fixed compensation). The fixed compensation strategy provided a fixed gravity torque calculated when the wrist was in the initial place. When the strategy without compensation was applied, subjects were asked to finish movements without the assistance of the cable-based robot. The weight of the split is 2 kg, thus when finishing movements without assistance, subjects were required to carry an extra 2 kg weight to guarantee the same experiment condition and to simulate the patients with arm weakness.

### Data analysis

RMSE between the target trajectory and the actual trajectory of wrist was used to evaluate movement accuracy.

(5) $$\text{RMSE=}\sqrt{\sum_{i\;=\;1}^{N}\left({\left(X_{a_{i}}-X_{t_{i}}\right)}^{2}+{\left(Y_{a_{i}}-Y_{t_{i}}\right)}^{2}+{\left(Z_{a_{i}}-Z_{t_{i}}\right)}^{2}\right)/N}$$

Where *i* refers to the sampling point, *X*_*a*_, *Y*_*a*_*i*__, *Z*_*a*_*i*__ refer to the actual values of 3D coordinates and *X*_*t*_*i*__, *Y*_*t*_*i*__, *Z*_*t*_*i*__ refer to the target values of 3D coordinates.

In order to evaluate the control abilities of arm, NJS of the actual trajectory during movement, was adopted to represent movement smoothness in 3D space (Hogan and Sternad, 2009).

(6) $$\text{NJS=}\sqrt{\frac{1}{2}\times \frac{T^{5}}{D^{2}}\times \int {\overset{...}{s(t)}}^{2}dt}$$

Where *t* refers to the actual time, *s*(*t*) refers to the position at the time of *t*, and *T, D* refer to the duration time, distance during the movement, respectively.

The MVR in each main motion direction is proposed for quantifying the relative velocity deviation in desired direction during movements tracking. It was assessed for movement efficiency, and was calculated as:

(7) $$MVR=\sum_{i}^{N}(V_{m_{i}}/\sqrt{V_{x_{i}}^{2}+V_{y_{i}}^{2}+V_{z_{i}}^{2}})/N$$

Where *i* refers to the sampling point, *V*_*m*_*i*__ refers to the velocity in the main motion direction at the point of *i*, and *V*_*x*_*i*__, *V*_*y*_*i*__, *V*_*z*_*i*__ refer to the velocity in X, Y, Z direction at the point of *i*, respectively. N refers to the total sampling points.

Envelopes of EMG signals were obtained after a full-wave rectification and a low-pass filter at 20 Hz. The muscle activation was calculated for each muscle and mean muscle activation of one muscle was the mean value during the whole sampling time in one tracking movement.

Two-way ANOVA (Analysis of Variance) as a statistical model was applied to test the main effect of compensation strategy (without, with fixed, and with position-varying gravity compensation), tracking direction (upward, forward, leftward, and rightward) and the interaction effect of these two factors on the RMSE, NJS, MVR, and mean muscle activation values. In the following, *post-hoc* Tukey tests as multiple comparisons were performed to test the difference on RMSE, NJS, and MVR. Paired *t*-tests were then utilized to compare RMSE, NJS, MVR, and mean muscle activation among the three compensation strategies in each direction. The significance level for all statistical tests was set at 0.05. All statistical analysis was conducted with SPSS version 22.0 (SPSS Inc., Chicago, IL).

## Results

**Figure 3:**
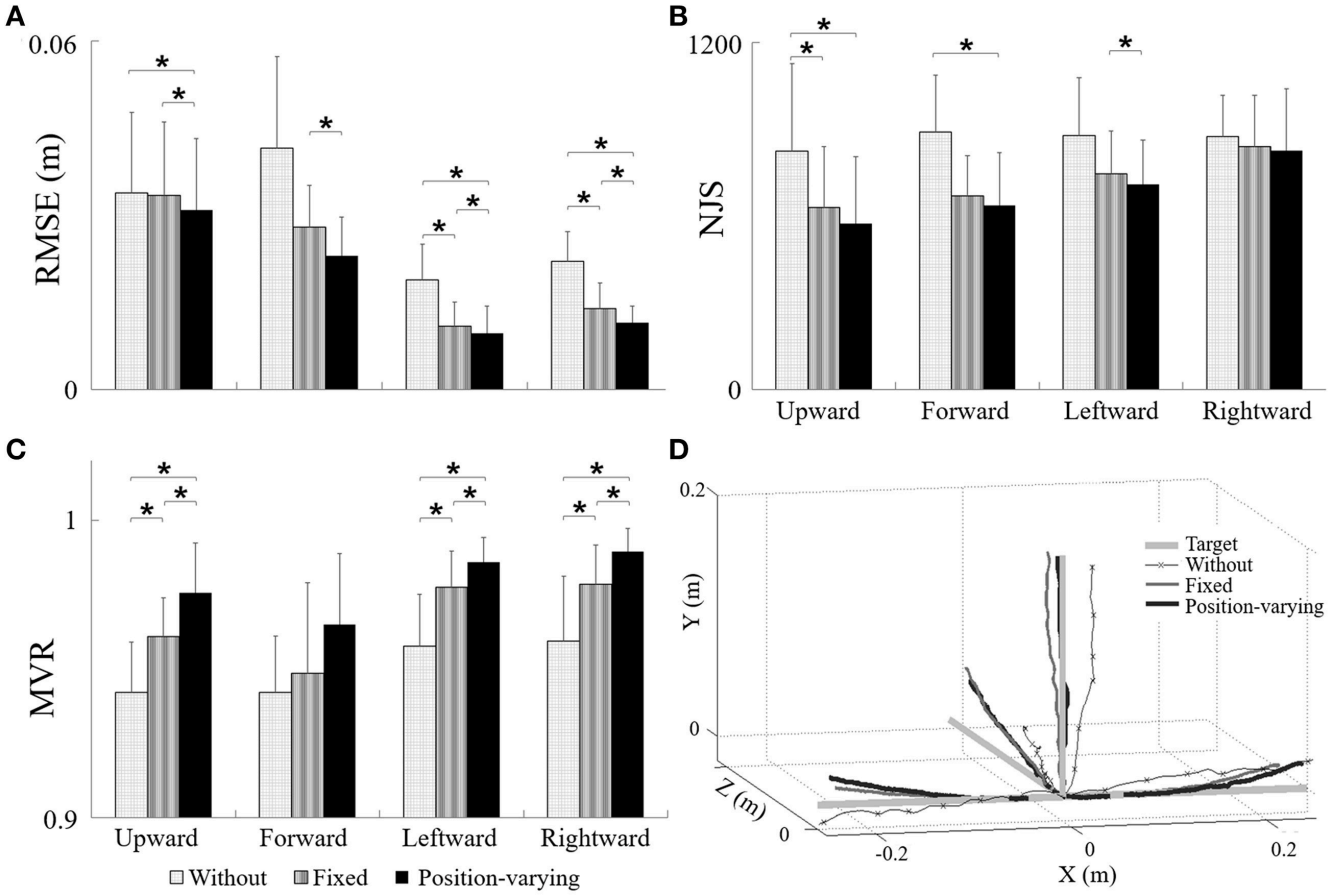
**The performance of (A)** RMSE, **(B)** NJS and **(C)** MVR during movements with different gravity compensation strategies, **(D)** the target, and actual trajectories during movements tracking with different gravity compensation strategies. ^*^Significant difference was found between two kinds of gravity compensation strategies (*p* < 0.05).

Figures 3A–C displayed RMSE (Figure 3A), NJS (Figure 3B), and MVR (Figure 3C) during movements tracking with different gravity compensation strategies. The results of ANOVA tests were shown in Table 1. As shown in Table 1, both compensation strategy and movement direction showed notable influences on RMSE (*P* < 0.05), NJS (*P* < 0.05), and MVR (*P* < 0.05). No significant interaction between compensation strategy and movement direction was found. The results showed that there were significant effects of compensation strategies in RMSE and NJS values represented by the following order: without compensation > fixed compensation > position-varying compensation, and MVR values represented by the following order: without compensation < fixed compensation < position-varying compensation. According to the *post-hoc* analysis, RMSE during upward and forward movements were remarkably higher than those during the leftward and rightward movements while MVR during upward and forward movements were significantly smaller than those during the leftward and rightward movements. Additionally, there was a significant decrease in NJS during upward movements compared with those during rightward movements. Based on paired *t*-test, RMSE of position-varying compensation strategy reduced significantly in leftward and rightward movements when comparing with those of without and fixed compensation strategy. In upward and forward movements, significant difference between the RMSE values of the position-varying and fixed compensation strategies were found. As for NJS, the significant difference between the position-varying and without compensation strategies during upward and forward movements and the significant difference between the position-varying and without compensation strategies during upward movement were found. All the MVR values with fixed and with position-varying gravity compensation were larger than those without gravity compensation. Comparisons of MVR between each two of three gravity compensation strategies showed significant difference in three directions: upward, leftward, and rightward. The actual trajectories in four movements from a same subject were plotted in Figure 3D.

**Table 1:** **The results for all factors involved in ANOVA tests**.

***F-*****value**		**Main effects**		**Interaction effect**
Outcome measures		Compensation Method	Target direction	Compensation Method × Target direction
		(DOF = 2)	(DOF = 3)	(DOF = 6)
RMSE		9.823 (*P* = 0.000)^*^	25.986 (*P* = 0.000)^*^	1.032 (*P* > 0.050)
NJS		6.625 (*P* = 0.002)^*^	2.872 (*P* = 0.042)^*^	0.495 (*P* > 0.050)
MVR		18.483 (*P* = 0.000)^*^	8.793 (*P* = 0.000)^*^	0.276 (*P* > 0.050)
Muscle activation	BIC	54.228 (*P* = 0.000)^*^	0.091 (*P* > 0.050)	0.842 (*P* > 0.050)
	TRI	4.415 (*P* = 0.020)^*^	0.928 (*P* > 0.050)	0.362 (*P* > 0.050)
	DA	49.943 (*P* = 0.000)^*^	7.628 (*P* = 0.000)^*^	1.622 (*P* > 0.050)
	DM	19.882 (*P* = 0.000)^*^	4.197 (*P* = 0.009)^*^	1.201(*P* > 0.050)
	DP	1.031 (*P* > 0.050)	2.594 (*P* > 0.050)	0.664 (*P* > 0.050)
	TRA	18.705 (*P* = 0.000)^*^	3.963 (*P* = 0.011)^*^	0.429 (*P* > 0.050)

* *Indicated significant difference (P < 0.05)*.

**Figure 4:**
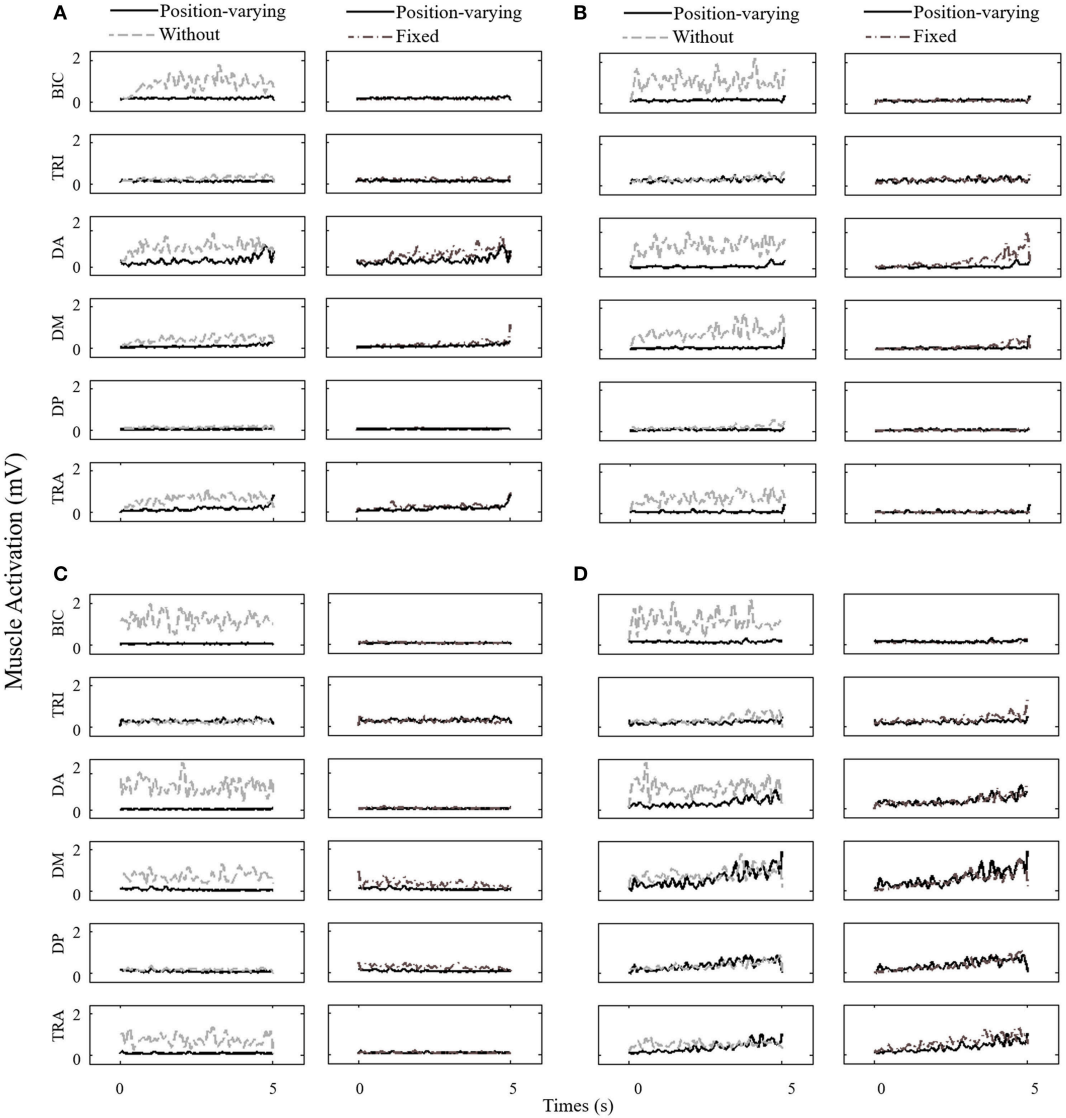
**EMG envelope time series of one subject for all muscles monitored during the study**. The data is shown for three gravity compensation strategies (without, fixed, and position-varying) and for the following six muscles: BRI, TRI, DA, DM, DP, and TRA. **(A)** Upward, **(B)** Forward, **(C)** Leftward, and **(D)** Rightward.

**Figure 5:**
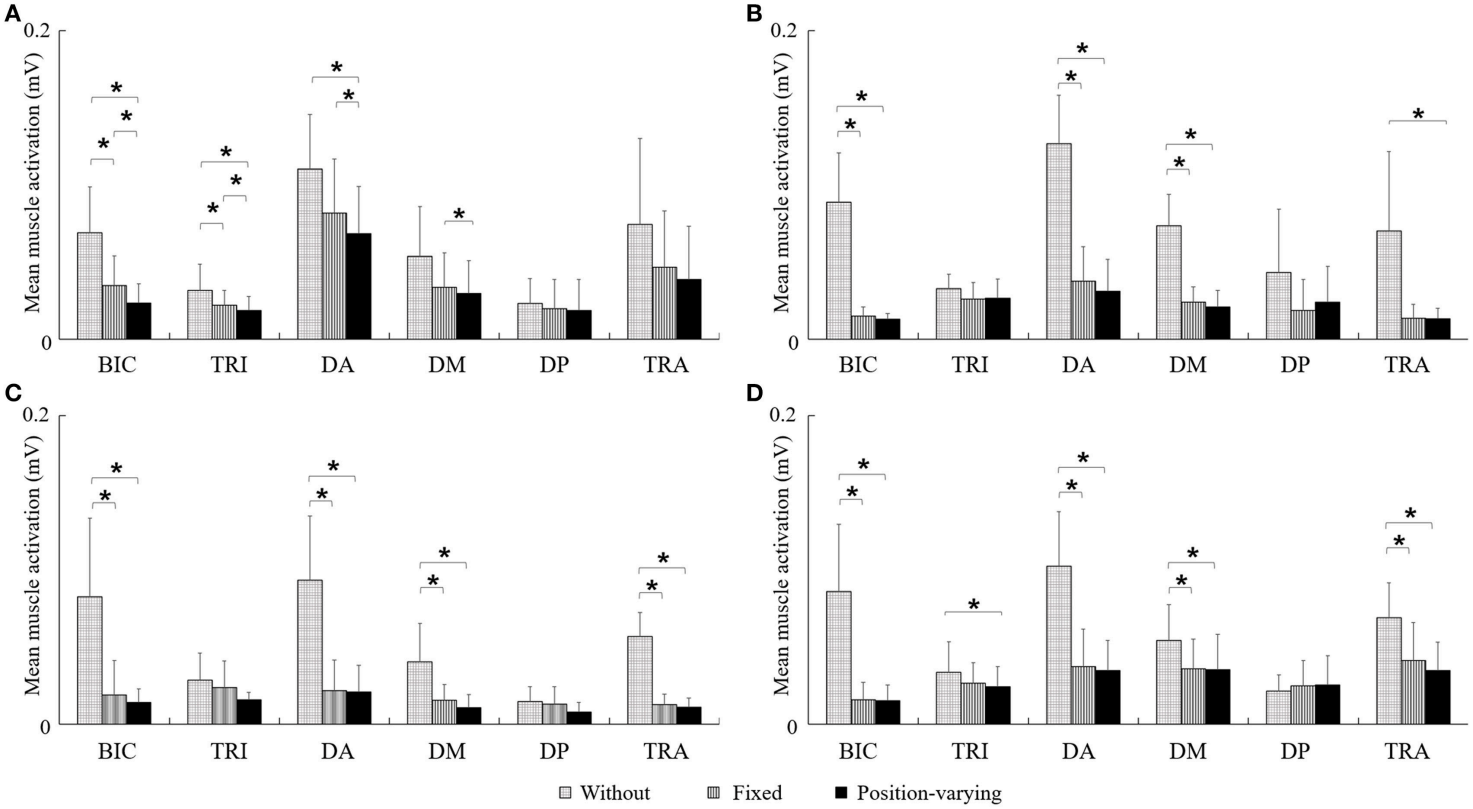
**The mean activation of six muscles during four direction movements with different gravity compensation strategies**. ^*^Significant difference was found between two kinds of gravity compensation strategies (*P* < 0.05). **(A)** Upward, **(B)** Forward, **(C)** Leftward, and **(D)** Rightward.

Figure 4 represented EMG envelope of the six muscles for one subject during the movements tracking with three different compensation strategies. The results indicated that when the subject moved upward, leftward, and rightward with gravity compensation, BIC, DA, DM, and TRA were less activated while he moved rightward, BIC and DA were less activated. Two-way ANOVA showed that effect of compensation strategy on mean muscle activation of BIC, TRI, DA, DM, and TRA (*P* < 0.05) together with effect of direction on mean muscle activation of DA, DM, and TRA were significant (*P* < 0.05). No significant interaction between compensation strategy and movement direction was found. In Figure 5, mean muscle activation of per muscle during four direction movements with different gravity compensation strategies were displayed. Compared with movements without compensation, the mean activation of some muscles during movements with position-varying compensation showed significant reductions [i.e., BIC and DA in all four directions, the mean activation of DM and TRA in three directions (forward, leftward, and rightward), and the mean activation of TRI in both upward and rightward]. The results revealed that the activation of BIC, TRI, DA, and DM with position-varying gravity compensation showed a significant decrease compared with fixed compensation during the upward movements.

## Discussion

The purpose of this study was to compare three different gravity compensation strategies by a cable-based robotic system. Kinematics parameters in terms of RMSE, NJS, and MVR together with mean muscle activations were applied to evaluate their movement performance. RMSE can reflect the general tracking accuracy, and is determined by sensory perception, motor planning and execution (Seppänen et al., 2013). The lower RMSE values with position-varying gravity compensation reflected improved tracking accuracy, which was in agreement with previous studies (Grimm et al., 2016). NJS reflected movement smoothness and was generally utilized to evaluate the arm control abilities (Adamovich et al., 2009). The significant decrease in NJS with compensation than without compensation were also reported by Coscia et al. who found significant difference in normalized jerk between without and with gravity compensation during arm reaching movements (Coscia et al., 2014). MVR is utilized to quantify the relative velocity deviation in desired direction during movements tracking. A similar parameter, normalized angular velocity ratio, was proposed by Kim et al. for assessing locomotion precision (Kim et al., 2014). The higher MVR values with position-varying gravity compensation indicated that subjects could more voluntarily participate in the desired movements. The effects of direction on movement kinematics were significant which was in accordance with previous studies in sagittal (Papaxanthis et al., 1998), frontal (d'Avella et al., 2008), or horizontal plane (Beer et al., 2004). Both RMSE and MVR could describe movement accuracy from different points of view, and higher RMSE during upward and forward movements were corresponding to lower MVR. There explanation of the result might be that, the variation of gravity torques during upward and forward movement might be larger.

There were significant reductions in main contributed muscle activations with position-varying and with fixed gravity compensation when compared with without compensation. Previous studies also found that BIC, DA, and TRA were the mainly contributed muscles for gravity support of upper limb, and the activations of these muscles also decreased significantly during movements in horizontal plane (Sabatini, 2002; Prange et al., 2009) and frontal plane (Kloosterman et al., 2010; Coscia et al., 2014). In addition, McCrea et al. proposed that DM was recruited if the primary anti-gravity muscles were not capable during upper limb movements (McCrea et al., 2005). The findings of this study were in accordance with the above-mentioned studies. Although, upper limb muscles activations were dependent on direction (Hughes et al., 2009), activations of DP and TRI were not affected by direction in this study. It might be explained by that they were not the main contributed muscles indicated by in low activations in the four directions. As reported, subjects could maintain satisfy dynamic criteria during movements such as optimization of motor command (Nakano et al., 1999) or energy expenditure (Soechting et al., 1995) by appropriately activating arm muscles. The reduction of the mean muscle activation due to gravity compensation indicated that subjects could put more focus on target-directed movements, and result in a larger range of motion or more repetitions of movements. Previous studies also reported subjects had larger range of motion on the horizontal panel with gravity compensation (Housman et al., 2009; Wang and Dounskaia, 2012). Furthermore, muscle activation is related to muscle forces(Lloyd and Besier, 2003), therefore, lower activation of muscles might reflect the robot is able to share the gravity loading, and muscle forces mainly focus on desired movement execution.

This study found that position-varying gravity compensation provided by the cable-based rehabilitation robot could improve man-machine cooperation movements in 3D working space in terms of RMSE, NJS, and MVR, which was the first explorative investigation on the position-varying gravity compensation to our knowledge. Moreover, since the position-varying gravity compensation strategy could reduce activations of anti-gravity muscles, it could assist patients with muscle weakness to perform rehabilitation training in clinic. The advantages of the position-varying compensation strategy are located on two aspects: firstly, the assistance is provided to counteract the effect of gravity; secondly, the assistance does not let the training in the passive way, and voluntary residual motor efforts can be focused on target-directed task. The reorganization in brains of subjects after stroke can be facilitated by active rehabilitation training (Cauraugh et al., 2000). In the future, more parameters should be adopted to clarify performance of the position-varying gravity compensation, and patients should be employed to explore the clinical effectiveness.

## Conclusion

The present study firstly explored the effect of position-varying gravity compensation strategy provided by a cable-based robot on kinematics and muscle activations in comparison with the other two gravity compensation strategies. The improvement in kinematics and less activated anti-gravity muscles with the position-varying gravity compensation indicated its potential in robot-aided rehabilitation therapy. More studies across pathologies with gender-matched subjects are needed to validate whether training with position-varying gravity compensation is clinically feasible and effective.

## Author contributions

YH and RS conceived and designed the study. YH and QY performed the experiments. YH and QY wrote the paper. RS reviewed and edited the manuscript. YH and QY contributed to the work equally and should be regarded as co-first authors. YC made a contribution to experiments. All authors had read and approved the manuscript.

## Funding

The project was supported by the National Natural Science foundation of China (Grant No. 61273359 and 91520201), the Guangdong Science and Technology Planning Project (Grant No. 2014B090901056 and 2015B020214003) and the Guangzhou Research Collaborative Innovation Projects (Grant No. 201604020108).

### Conflict of interest statement

The authors declare that the research was conducted in the absence of any commercial or financial relationships that could be construed as a potential conflict of interest.
